# 24-month patient-reported outcomes for a novel lumbar total joint replacement

**DOI:** 10.1016/j.xnsj.2025.100747

**Published:** 2025-06-13

**Authors:** Ahilan Sivaganesan, Marissa Koscielski, Ashmal Sami Kabani, J. Alex Sielatycki, Jeffrey Goldstein, Brady Riesgraf, Craig Humphreys, Scott Hodges

**Affiliations:** a Department of Neurosurgery, Thomas Jefferson University and Jefferson Hospital for Neuroscience, 909 Walnut Street, Philadelphia, PA, United States; b Department of Spine Surgery, Hospital for Speciality Surgery at Naples Comprehensive Health Center, 11190 Health Park Blvd Ste 2102, Naples, FL, United States; c University of Cincinnati College of Medicine, 3230 Eden Ave, Cincinnati, OH, United States; d Steamboat Orthopedic and Spine Institute, 705 Marketplace Plaza, Steamboat Springs, CO, United States; e Department of Orthopaedic Surgery, NYU Langone Health, 333 E 38th St., 6th Floor, New York, NY, United States; f 3Spine, 801 Broad St., 6th Floor, Chattanooga, TN, United States; g Kenai Spine, 240 Hospital Pl #104, Soldotna, AK, United States; h Spine Motion Specialists, 801 Broad St., 2nd Floor, Chattanooga, TN, United States

**Keywords:** 3Spine, Degenerative disc disease, Lumbar fusion, Lumbar total joint replacement (TJR), Motion-sparring, ODI score

## Abstract

**Background:**

Lumbar fusion remains a prevalent treatment for degenerative conditions; however, its limitations have sparked interest in alternative motion-sparing procedures. Our study evaluates 24-month postoperative patient-reported outcomes from an OUS pilot clinical study on a novel lumbar total joint replacement (TJR) for degenerative conditions.

**Methods:**

Data was collected from 63 patients, of which 56 patients fulfilled the inclusion criteria. Self-reported measures collected for this study are Oswestry Disability Index (ODI), Numeric Rating Scale (NRS), Minimal Symptom State (MSS), Minimal Clinical Important Difference (MCID), Substantial Clinical Benefit (SCB). This retrospective analysis of prospective, IRB-approved collected data reports 24 month patient-reported outcomes on a cohort receiving lumbar TJR. The cohort includes skeletally mature individuals who underwent lumbar TJR at 1-3 Lumbar levels (L1–S1) between 2008 and 2019. Conservative treatment was mandatory for at least 3 months unless facing a neurologic emergency or intractable pain. Descriptive analysis was performed for continuous variables and frequencies were calculated for categorical variables.

**Results:**

63 patients were treated with lumbar TJR and electively participated in data collection after 12 months. 56 patients, with age ranging from 19 to 82 years, and 93 levels were treated with lumbar TJR at 1-3 lumbar levels and had complete follow-up data at 12 and 24 m. No device-related adverse events were reported during the 12-to-24-month follow-up window. At 24 months, patients exhibited sustained clinical improvement in back pain, leg pain, and disability scores, similar to the 12-month observations. An overall improvement in Minimal Clinically Important Difference (MCID) was also noted.

**Conclusions:**

Our study shows consistent improvement in PROs, indicating the clinical improvement of lumbar TJR at both the 12-month and 24-month follow-up points, compared to baseline. Acknowledging limitations, including the lack of comparative data with standard of care, these findings suggest that TJR may be a treatment option for indicated lumbar degenerative pathologies.

## Introduction

Lumbar fusion has long been the mainstay of surgical treatment for many common degenerative spinal conditions. Effective motion-preserving alternatives, such as anterior disc replacement (ADR), are available for individuals affected by isolated degenerative disc disease, albeit within constrained parameters [1,2]. There is no doubt that there will likely always be a major role for lumbar fusion for patients with spinal deformity, gross instability, infection, trauma, and certain neurologic emergencies. However, lumbar fusion does carry an inherent risk of re-operation due to adjacent segment disease, hardware-related complications, malalignment, and pseudoarthrsis [[3], [4], [5], [6], [7], [8], [9], [10], [11], [12], [13], [14]]. Spinal fusions, and the costs of subsequent surgeries, exert a notable financial impact on the American healthcare system, with the annual cost of lumbar spinal fusions in the United States surpassing $10billion in 2015, an increase of 138% over 2014 [15,16]. In light of all this, there is clinical and societal need for a widely applicable motion-preservation procedure that can address common degenerative pathologies which currently require fusion.

In 2021, we published a comparative analysis contrasting a newly introduced lumbar total joint replacement (TJR) prosthesis with 1-2 level transforaminal interbody fusion (TLIF) as interventions for degenerative conditions [17]. In our study, we documented statistically significant improvements in lumbar-related disability (Oswestry Disability Index—ODI), back pain, and leg pain for both the fusion and lumbar TJR groups from baseline to 3 months. Notably, the lumbar TJR group exhibited sustained improvements in ODI, Numeric Rating Scale (NRS) for back pain, and NRS for leg pain from 3 months to 12 months, whereas no additional improvement was observed in the fusion cohort during this period. In this report, we aim to present the 24-month patient-reported outcomes (PROs) for individuals undergoing lumbar TJR and provide a comprehensive account of adverse events observed.

## Material and methods

### Study design

This retrospective analysis of IRB-approved prospectively collected data (WIRB 1174471) reports the 24-month PROs for a novel lumbar TJR from a pilot clinical study. Informed consent was obtained from every patient before the procedure and enrollment in this data collection study. The cohort encompasses skeletally mature individuals who underwent lumbar TJR at one to 3 lumbar levels (L1–S1) between 2008 and 2019, due to symptomatic lumbar degeneration, including spondylolisthesis (not exceeding grade 1), recurrent disc herniation, and adjacent segment disease, with or without foraminal/recess stenosis, confirmed by X-Ray and advanced imaging (MRI or CT). Patients were required to undergo conservative (nonoperative) treatment for at least 3 months (including physical therapy, invasive cortisone injections, and anti-inflammatory medications) unless faced with a neurologic emergency or intractable pain.

**Fig. 1:**
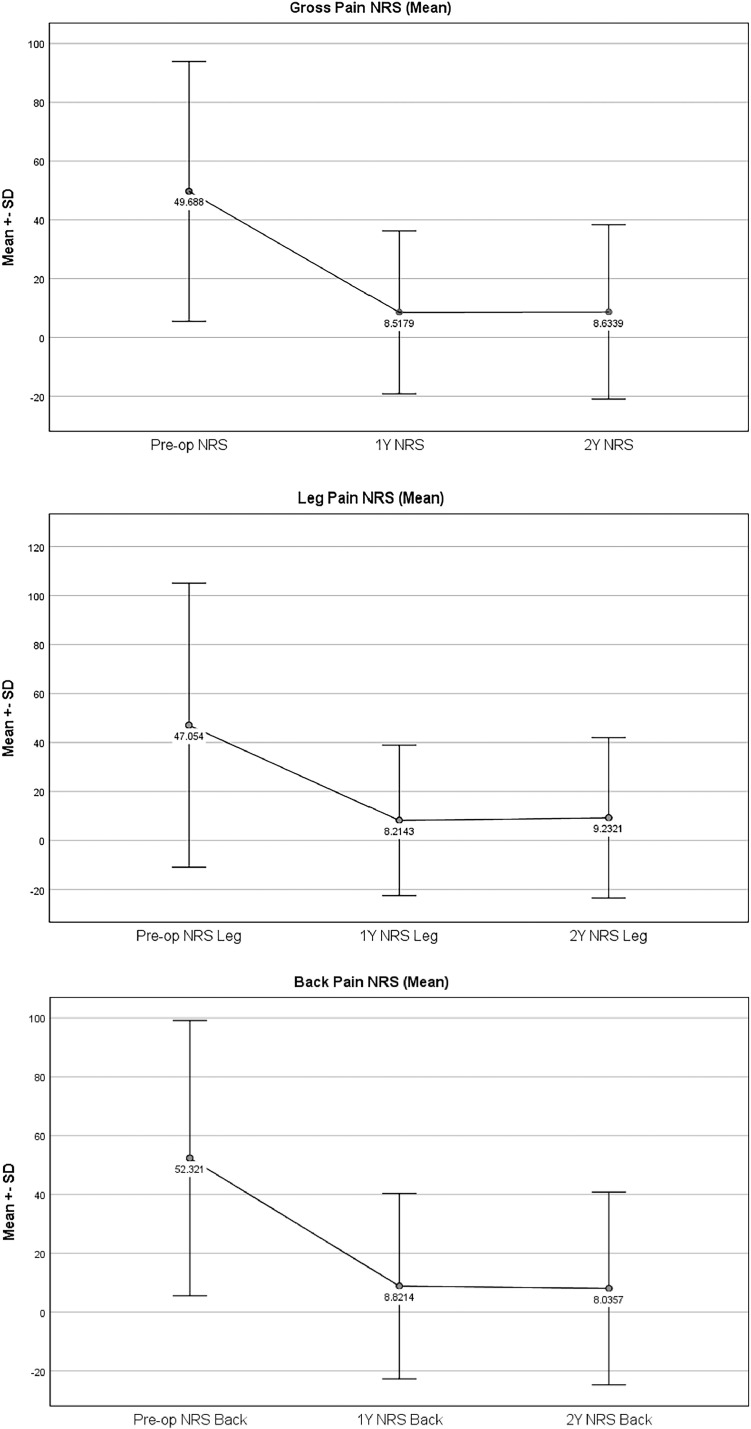
Mean ODI and NRS at Pre-op, 12-month, and 24-month intervals.

**Fig. 2:**
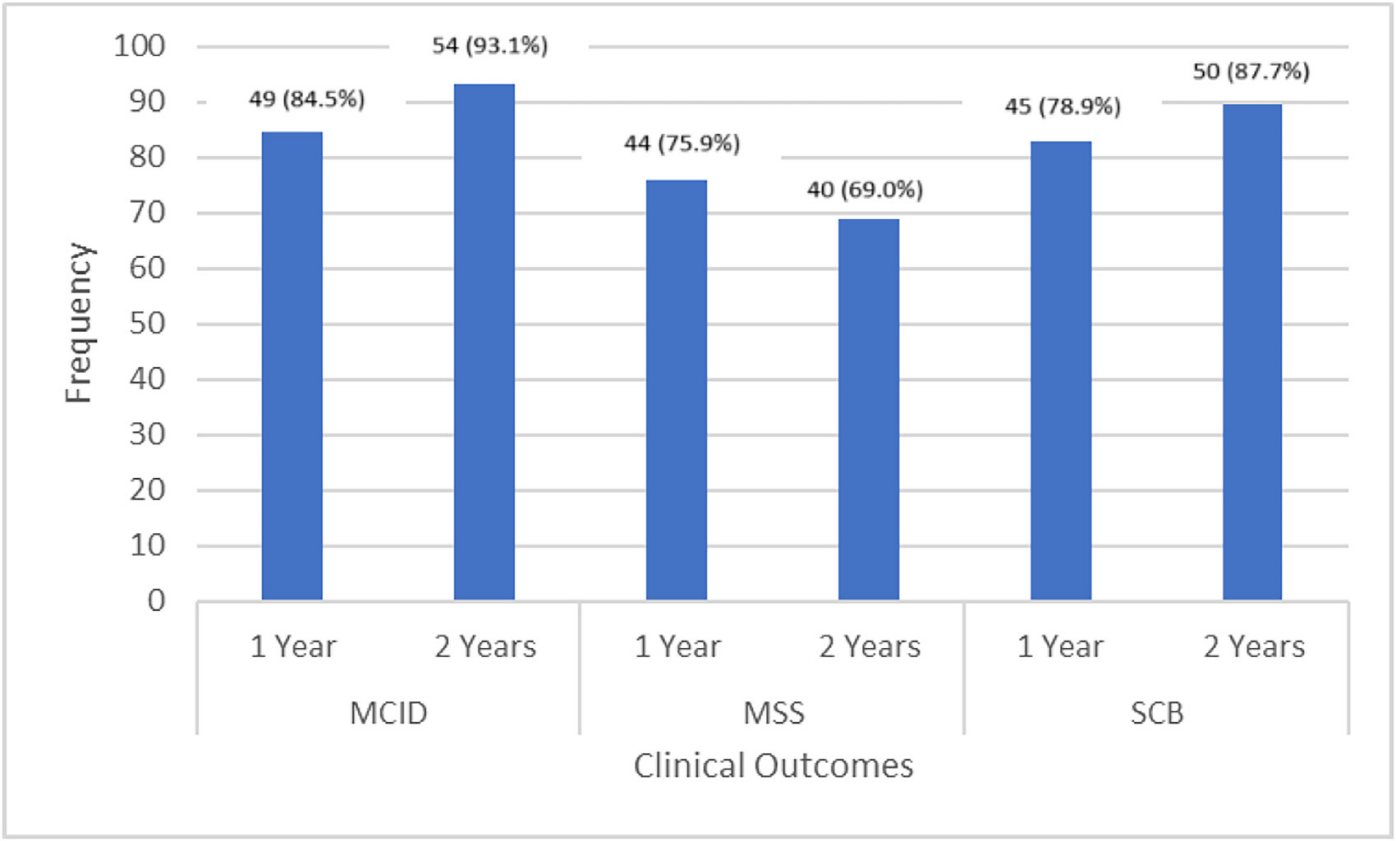
Frequency of patients that achieved MCID, MSS and SCB at 12- and 24-month intervals.

Lumbar TJR exclusion criteria encompassed individuals with spondylolisthesis exceeding grade 1, solid fusion at the indicated level, tumors, infections, severe deformities, osteoporosis, or trauma. Patients with baseline and 12-month data could electively choose to participate in annual, long-term follow-up. Patients with complete outcomes and safety data (adverse events) at baseline, 12-months, and 24-months, as of April 2023, were included in this analysis. Outcomes and safety data from patients with additional long-term follow-up were also reported (Fig. 1, Fig. 2).

### Lumbar TJR implant

**Fig. 3:**
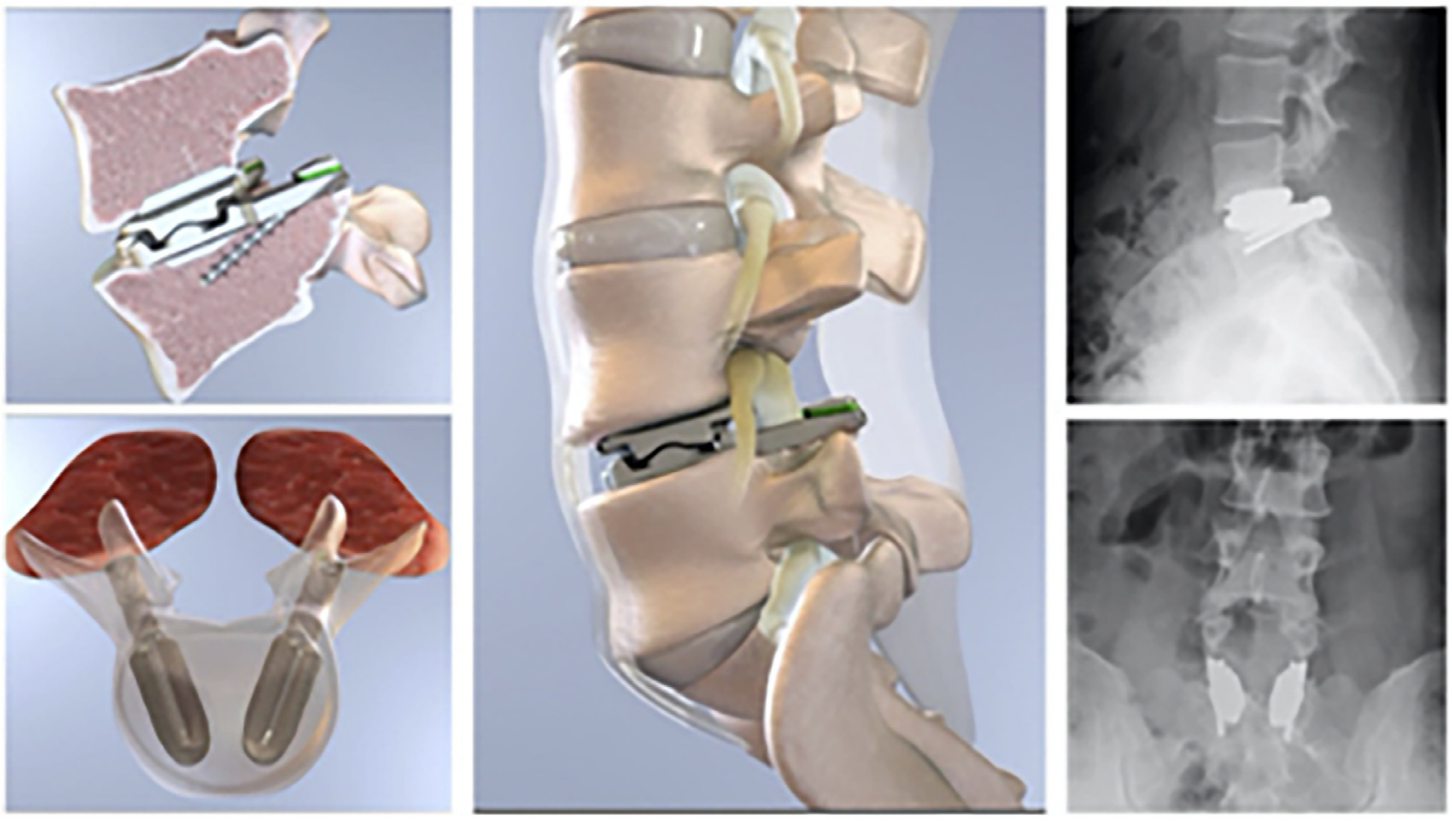
Illustration of Lumbar TJR and radiograph of implant in coronal and sagittal plane at L4–L5 [17].

Details regarding the implant and insertion technique can be found in the 12-month publication, as well as in Appendix A. The lumbar TJR constitutes a reconstruction of the lumbar motion segment, inserted through a bilateral transforaminal lumbar interbody fusion (TLIF) approach subsequent to laminectomy, bilateral facet removal, and comprehensive discectomy, aiming for extensive central and bilateral decompression of neural elements [17]. Endplate preparation involves achieving parallel endplates through a pedicle - vertebral body osteotomy and cutting coplanar keels along the convergent trajectory of the pedicle in both the cephalic and caudal vertebral bodies Fig. 3. Length and height trials and intraoperative imaging help determine the appropriate prosthesis size. At each respective level, 2 prostheses are then implanted along the axis of the corresponding pedicle. The preservation of the integrity of the cephalad endplate, lateral annulus, and anterior complex, including the anterior longitudinal ligament and anterior portion of the anterior annulus, is prioritized. The design of the lumbar TJR is intended to emulate the function of both the disc and facet joints, providing stabilization subsequent to the reconstruction of the treated motion segment(s).

### Clinical outcome measures

The ODI, NRS for back pain, and NRS for leg pain were systematically documented preoperatively and 12, and 24 months postoperatively. The ODI and NRS were selected for their widespread validation in lumbar spine populations, reflecting functional and pain domains essential to assessing surgical efficacy. ODI serves as the key metric for evaluating functional status in these patients. This assessment comprises ten self-reported, pain-related inquiries related to everyday activities. Scores are expressed as percentages on a 100-point scale, with ≤20% denoting minimal disability, 21%–40% indicating moderate disability, 41%–60% reflecting severe disability, 61%–80% signifying being crippled, and 81%–100% representing a bedridden state [18]. Back and leg pain were independently assessed using a 0 to 10-point NRS, where higher scores correspond to increased pain severity [19,20]. Patients were asked to rate their pain on a scale of 0 to 10, with 0 signifying no pain and 10 signifying the worst pain imaginable.

ODI and NRS scores were used to calculate the Minimal Symptom State (MSS) and the ODI scores were used to define the Minimal Clinical Important Difference (MCID) and Substantial Clinical Benefit (SCB) at 12-months and 24-months after surgery. Implant-related adverse events were also tallied. The MSS was defined as achieving an ODI score below 20% along with NRS back and leg pain scores less than 2, per Crawford et al. [11]. The MCID was operationally defined as a 30% reduction in the overall ODI score relative to preoperative scores. This threshold has been shown to be superior to point-change MCID thresholds and has been validated in patients undergoing lumbar spine surgery [21]. SCB was also utilized as an 18-point reduction in the overall ODI score from baseline [22].

### Data analyses

All data analyses were conducted on R version 4.3.2 [21]. Descriptive statistics, encompassing mean (M) and standard deviation (SD) for continuous variables, and frequency and percentage for categorical variables, were computed. Patient demographics, operative variables, and preoperative PROs were subjected to comparison between patients at 12-month and 24-month time-points.

Mean values of the ODI and NRS scores were graphically represented for preoperative, 12-month and 24-month intervals postsurgery. Proportions of patients achieving the MCID, SCB, and MSS at each time point was also calculated. Statistical significance was determined through Fisher's exact test for categorical variables and the independent samples t-test for continuous variables.

## Results

Baseline characteristics of the study population are listed in Table 1. A total of 63 patients underwent lumbar TJR surgery between 2008 and 2019 at 3 clinical sites and electively participated in longer-term safety and outcomes data collection after the 12-month timepoint. Of these eligible patients, 56 had complete baseline, 12-month, and 24-month data. Two additional patients had complete baseline and 24-month data.

**Table 1:** Baseline characteristics.

Characteristic	*M* ± SD/N (%)
Sex	
Men	40 (71.4%)
Women	16 (28.6%)
Age (years, min–max)	19–82
BMI	28.00 ± 3.88
Level L1–L2	-
Level L2–L3	3 (5.2%)
Level L3–L4	20 (34.5%)
Level L4–L5	42 (72.4%)
Level L5–S1	28 (48.3%)
Number of Levels Treated	
1	27 (46.6%)
2	27 (46.6%)
3	4(6.9%)
Blood Loss (mls)	279.64 ± 144.78

Of all the patients treated with lumbar TJR from this pilot cohort, 7 patients were removed prior to the 12 m timepoint due to loss of follow-up (1), revision surgery (3, reported in our 2021 paper) [17], or an unrelated medical condition (2).

56 patients with a ages of 19 to 82 years (71.4% of which were men) and 93 levels were treated with lumbar TJR at 1–3 lumbar levels with complete follow-up data at 12-months and 24-months. 46.6% were single level lumbar TJR cases and 53.4% of patients received multilevel surgeries. L4–L5 (72.4%) was the most common treated level, followed by L5–S1 (48.3%), L3–L4 (34.5%), L2–L3 (5.2%), and L1–L2 (0%).

### Patient reported outcomes

Mean ODI improved at each interval (46.82 at baseline, 10.19 at 12 months, and 8.70 at 24 months). NRS for back and leg pain improved at each time point until the 12-month mark and slightly increased at 24-month follow-up. Mean preoperative back pain improved from 52.32 to 8.82 at 12-month, and to 8.04 at the 24-month mark. Mean leg pain improved from 47.05 preoperatively to 8.21 at 12-months, and then increased to 9.23 at the 24-month mark as seen in Table 2.

**Table 2:** Patient reported outcomes at preoperative, 3 months, 6 months, 12 months, and 24 months (N, Mean ± SD).

Patient reported outcomes	N	Mean	SD
ODI			
Preoperative	56	46.82	18.53
12 months	56	10.19	11.43
24 months	56	8.70	10.09
Leg pain (NRS)			
Preoperative	56	47.05	28.99
12 months	56	8.21	15.36
24 months	56	9.23	16.39
Back pain (NRS)			
Preoperative	56	52.32	23.39
12 months	56	8.82	15.77
24 months	56	8.04	16.37

MSS was obtained by 75.9% of patients at 12-months, and 69.0% of all patients maintained MSS at 24-months, documented in Table 3. Improvement was seen in MCID for 84.5% of patients at 12-months to 93.1% of patients at 24-months. 78.9% of patients demonstrated an 18-point reduction from baseline in ODI at 12-months (SCB), and this improved to 87.7% of patients at the 24-month mark. No device or procedure-related adverse events were reported during the 12-to-24-month follow-up window.

**Table 3:** Clinical outcome metrics tabulated at 3-months, 6-months, 12-months, and 24-months.

Clinical outcome measures	N (%)
MCID (30% reduction in baseline ODI)	
12 months	49 (84.5%)
24 months	54 (93.1%)
MSS (ODI <20, NRS back <20, NRS leg <20)	
12 months	44 (75.9%)
24 months	40 (69.0%)
SCB (18-point reduction from baseline in ODI)	
12 months	45 (78.9%)
24 months	50 (87.7%)

### Adverse events

Surgical and device-related adverse advents that occurred between baseline and 12-months were reported in our 2021 study [17]. No device or procedure-related adverse events occurred during the 12-to-24-month follow-up period.

## Discussion

### Summary of findings

In this study, we report long-term 24-month clinical outcomes for a cohort of patients who underwent the lumbar TJR procedure. These patients demonstrated sustained clinical improvement in the back pain, leg pain, and disability scores that were observed at the 12-month postoperative mark. There was an overall improvement in MCID rates from the 12-month time point to 24-months. There were no additional procedure-related adverse events that were reported during this additional follow-up period.

In addition, exploratory raw analysis at 36-month interval presented a mean ODI of 9.87, gross NRS of 9.91, mean leg pain of 11.71 and mean back pain of 8.12. Preliminary analysis at 36-month interval present improvement in clinical outcome metrics in comparison to 24-month interval with 72.5% achieving MSS, 95.0% in MCID, and 77.6% patients obtained SCB. 89.66% of 51 patients with completed 36-month follow-up, demonstrated sustained clinical outcomes with an average a 1-point improvement in back pain, 2-point decline in Leg Pain, and a 1-point variance in disability at the 36-month mark as compared to 24-months. These interim results suggest that there may be sustained clinical improvement 36 months after lumbar TJR.

### Prior study

In 2021, we reported the statistically significant benefits for patients undergoing lumbar TJR, as compared to a propensity-matched cohort of TLIF patients, 12 months after surgery. In this study, the lumbar TJR cohort demonstrated improved rates of MCID and SCB, and only slightly reduced MSS, at the 24-month mark as compared to the 12-month mark. This study suggests that the clinical improvement observed in the lumbar TJR cohort continues after 12-months.

### Implications

TLIF stands as a historical mainstay for the treatment of many degenerative lumbar diseases [22]. Lumbar fusion has traditionally enabled a wide neural decompression for both lateral recess and the neural foramina, as well as allowed for stabilization of hyper-mobile spinal segments [23,24]. Despite its effectiveness, the TLIF procedure is not without its associated complications, which have become a focus of study by various researchers. Fusion-associated sequelae, such as cage subscience, can potentially lead to intervertebral collapse, recurrence of sciatica, elevated reoperation rates, and poor clinical efficacy [25]. Common causes for revision surgery following lumbar fusion are worsening of primary etiology or progressive degenerative disease; mechanical failure; stenosis or infection [26]. With no device-related adverse events occurring in the 12-to-24-month period following lumbar TJR, this pilot study suggests that lumbar TJR may have the capability to reduce these potential complications.

Rising revision rates and worsening ODI scores 12 months after fusion surgery, reported in the 2021 literature, highlight the need for motion-sparing alternatives to fusion for common degenerative pathologies [17]. Although limited in indication, anterior disc replacements and facet replacements have demonstrated improved patient reported outcomes at 24 months over fusion [27]. Most recently the TOPs Facet Replacement system reported improving ODI scores through 24 months, which was statistically superior to its comparative cohort in an FDA clinical trial [28]. Given the concurrence of facet, neural, and disc pathologies in common degenerative conditions, there is a need for a motion-preserving technology that is able to address all 3 components. The design of the lumbar TJR is intended to replace the function of the facet joints and the disc following decompression and reconstruction of the motion segment.

### Limitations

This study has notable limitations. Firstly, a comparative analysis between TLIF and lumbar TJR could not be conducted due to the limited availability of 24-month TLIF data in existing spine registries. Despite this constraint, we believe that reporting the accessible longer-term clinical and safety follow-up data on lumbar TJR is important for the ongoing exploration of this technology. Additionally, all patients included in this report were enrolled in the study prior to the Food and Drug Administration Investigational Device Exemption study on the MOTUS lumbar TJR. This suggests a potential confirmation bias in these patients, as they actively seek alternatives to lumbar fusion.

The MOTUS device is currently undergoing FDA clinical trials to investigate the safety and efficacy of the lumbar TJR procedure at a single segment in the lumbar spine.

## Conclusions

Our study provides an extended longitudinal evaluation of the clinical outcomes 24 months after lumbar TJR surgery. Despite challenges in conducting a comparative analysis with TLIF due to limited available control data, our findings underscore the sustained clinical improvement observed in patients undergoing lumbar TJR.

The results show a durable improvement in PROs, including reduced disability scores, back and leg pain at various time intervals. Notably, the study demonstrates an improvement in MSS and MCID metrics, when comparing the 24-month mark to the 12-month mark, indicating continued clinical gains for lumbar TJR in the long term.

Crucially, our investigation highlights the absence of additional device-related adverse events during the 12-to-24-month follow-up window. Furthermore, the interim results extending to the 36-month follow-up time point suggest there may be sustained clinical benefits with minimal decline in outcomes, highlighting the potential longer-term advantages of this motion-sparing lumbar TJR.

While early findings from this pilot study suggest promising long-term outcomes with lumbar TJR, randomized or comparative studies are needed to validate these findings against standard-of-care lumbar fusion.

In conclusion, our study contributes valuable insights to the evolving landscape of lumbar spine surgery, highlighting the lumbar TJR as a motion-preserving alternative to lumbar fusion.

## Declaration of competing interest

The authors declare that they have no known competing financial interests or personal relationships that could have appeared to influence the work reported in this paper. All disclosures are listed in the Footnotes.

## Appendix Supplementary materials

Image, application 1
